# Extracorporeal photopheresis as an immunomodulatory treatment modality for chronic GvHD and the importance of emerging biomarkers

**DOI:** 10.3389/fimmu.2023.1086006

**Published:** 2023-02-17

**Authors:** Ines Bojanic, Nina Worel, Carolina P. Pacini, Georg Stary, Agnieszka Piekarska, Aisling M. Flinn, Kimberly J. Schell, Andrew R. Gennery, Robert Knobler, João F. Lacerda, Hildegard T. Greinix, Drazen Pulanic, Rachel E. Crossland

**Affiliations:** ^1^ Department of Transfusion Medicine and Transplantation Biology, University Hospital Center Zagreb, Zagreb, Croatia; ^2^ School of Medicine, University of Zagreb, Zagreb, Croatia; ^3^ Department of Transfusion Medicine and Cell Therapy, Medical University of Vienna, Vienna, Austria; ^4^ Hematology and Transplantation Immunology, Instituto de Medicina Molecular, Faculdade de Medicina da Universidade de Lisboa, Centro Hospitalar Universitário Lisboa Norte, Lisbon, Portugal; ^5^ Department of Dermatology, Medical University of Vienna, Vienna, Austria; ^6^ CeMM Research Center for Molecular Medicine of the Austrian Academy of Sciences, Vienna, Austria; ^7^ Ludwig Boltzmann Institute for Rare and Undiagnosed Diseases, Vienna, Austria; ^8^ Department of Hematology and Transplantology, Medical University of Gdansk, Gdansk, Poland; ^9^ Translational and Clinical Research Institute, Faculty of Medical Sciences, Newcastle University, Newcastle upon Tyne, United Kingdom; ^10^ Paediatric Stem Cell Transplant Unit, Great North Children’s Hospital, Newcastle upon Tyne, United Kingdom; ^11^ Division of Hematology, Medical University Graz, Graz, Austria; ^12^ Division of Hematology, Department of Internal Medicine, University Hospital Center Zagreb, Zagreb, Croatia

**Keywords:** chronic GvHD, extracorporeal photopheresis, biomarker, immunomodulation, hematopoietic stem cell transplantation

## Abstract

Haematopoietic stem cell transplantation (HSCT) is the treatment of choice for malignant haematological diseases. Despite continuous improvements in pre- and post-transplantation procedures, the applicability of allo-HSCT is limited by life-threatening complications such as graft-versus-host disease (GvHD), engraftment failure, and opportunistic infections. Extracorporeal photopheresis (ECP) is used to treat steroid resistant GvHD with significant success. However, the molecular mechanisms driving its immunomodulatory action, whilst preserving immune function, require further understanding. As ECP is safe to administer with few significant adverse effects, it has the potential for earlier use in the post-HSCT treatment of GvHD. Thus, further understanding the immunomodulatory mechanisms of ECP action may justify more timely use in clinical practice, as well as identify biomarkers for using ECP as first line or pre-emptive GvHD therapy. This review aims to discuss technical aspects and response to ECP, review ECP as an immunomodulatory treatment modality for chronic GvHD including the effect on regulatory T cells and circulating vs. tissue-resident immune cells and consider the importance of emerging biomarkers for ECP response.

## Introduction

Allogeneic haematopoietic stem cell transplantation (HSCT) is a potentially curative treatment for a variety of haematologic and non-haematologic disorders. Despite improvement in HLA typing, optimization in conditioning regimens and current immunosuppressive protocols, acute and chronic graft-versus-host-disease (GvHD) remain the most important non-relapse post-HSCT complications (1, 2). Chronic graft-versus-host disease (cGvHD) occurs in approximately 30-70% of patients and remains the leading cause of non-relapse mortality (NRM) in patients surviving longer than two years after HSCT, affecting both quality of life and long-term treatment outcome (2, 3). The clinical management of patients with extensive cGvHD is challenging, due to the wide variability of disease manifestations, clinical course, infectious complications, and treatment related toxicity (4). Currently, the first-line therapy for chronic GvHD (cGvHD) relies on the administration of corticosteroids with a calcineurin inhibitor. However, its lack of efficacy in some patients, the broad immunosuppression induced in the patient, and significant adverse effects stresses the need for new alternative therapies. Extracorporeal Photopheresis (ECP) is a valuable second-line treatment for a subset of patients with steroid-refractory cGvHD, particularly those with severe cutaneous involvement (5–8).

Immunomodulatory ECP therapy is used in several inflammatory or autoimmune conditions in addition to, or instead of conventional immunosuppression. The advantage of immunomodulatory therapy is manipulation of the immune system leading to resolution of the underlying diseases process, but with preservation during treatment of beneficial aspects of immunity, such as anti-viral or anti-tumour activity. However, the molecular mechanisms driving ECP therapy’s unique immunomodulatory action, whilst still preserving immune function, are not completely understood. The mode of ECP action at a cellular or molecular level is likely to be the same, as the ECP process does not differentiate which disease is being treated – however, the outcome may be different, depending on the disease, and disease-status of the patient.

## Techniques and considerations for performing ECP

### Overview of the ECP procedure

**Figure 1:**
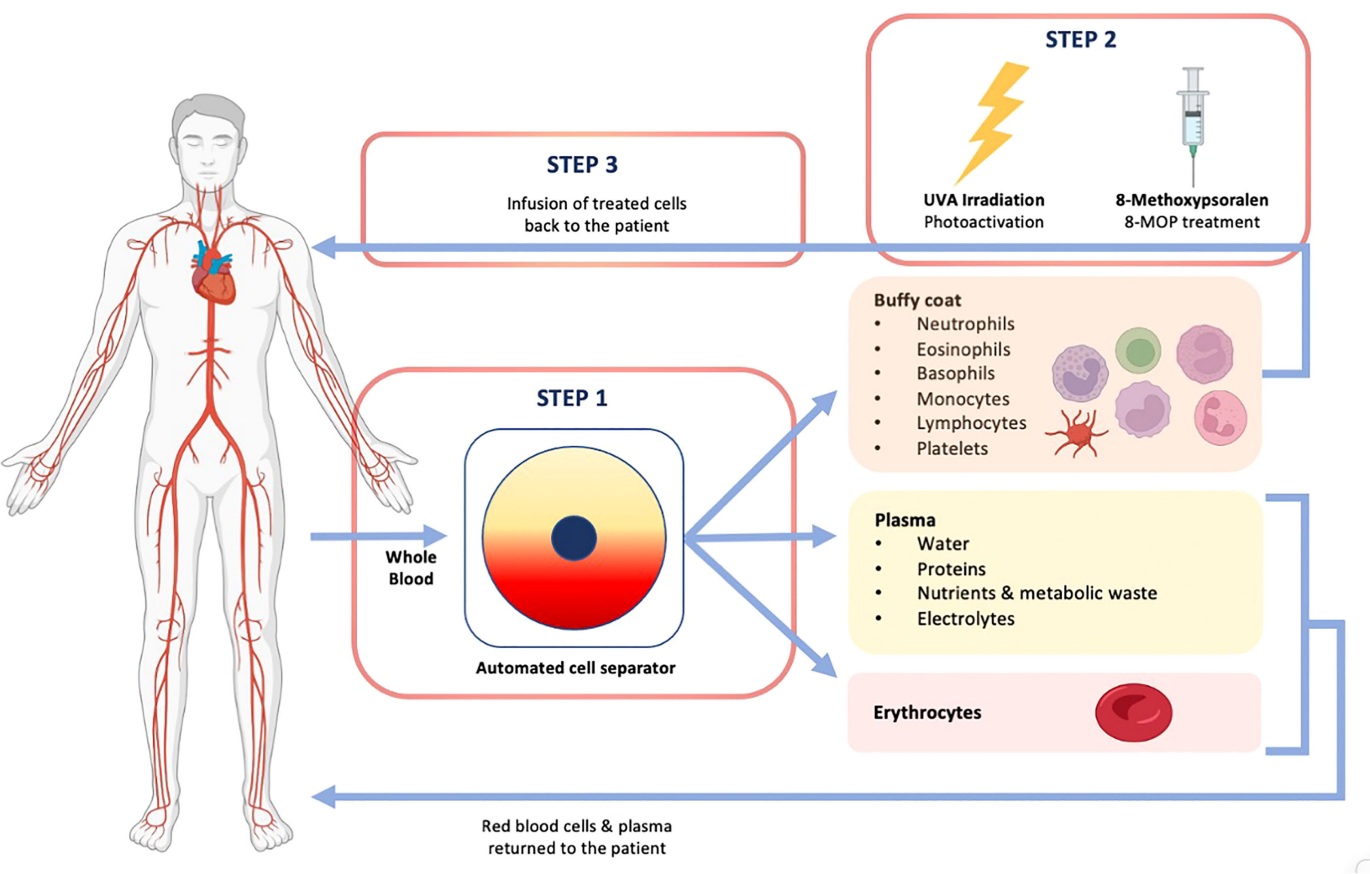
Overview of the ECP procedure.

The ECP procedure involves three steps and takes between two to four hours (**Figure 1**). Firstly, white blood cells (WBC) are automatically separated and collected from peripheral blood during a leukapheresis procedure. Secondly, collected cells are photoactivated with 8-methoxypsoralen (8-MOP), making them sensitive to ultraviolet A (UVA) light irradiation. 8-MOP is a naturally occurring inert substance that after irradiation with UVA, binds covalently to DNA, cell membranes, and proteins. This leads to apoptosis of treated cells and other cytotoxic effects (9). The suspension of cells and 8-MOP is exposed to UVA light (320-400 m wavelength). Thirdly, treated cells are reinfused into the patient. ECP treatment is traditionally administered in cycles, which are repeated with varying intervals according to the severity of disease symptoms (10). Each cycle consists of two sessions of ECP on two consecutive days, with clinical assessment for response every week in acute GvHD (aGvHD) and every 8–12 weeks in cGvHD. Cycles are tapered and tailored as per indication, institution, and clinical response.

### Techniques for performing ECP

Currently there are two conventional techniques for ECP treatment, open “off-line” and closed “in-line” techniques, classified based on the type of device used. The “in-line” technique uses a single-unit system in which the whole treatment is performed sequentially in extracorporeal circulation as a closed, one-step procedure. In the “off-line” technique, the target cells are collected by a standard apheresis device and after addition of photosensitiser 8-MOP, irradiated using an external UVA light irradiator device. Most centres perform ECP treatment using the approved closed system, driven by reduced risk of infection and infusion errors (11).

The “in-line” ECP procedure can be performed on the Therakos CELLEX Photoapheresis System (Mallinckrodt Pharmaceuticals, NJ, USA). The device uses a centrifugal force to separate and collect the mononuclear fraction (buffy coat) containing lymphocytes and monocytes, while the red cells and plasma are returned to the patient. The buffy coat remains in the system where it is treated with 8-MOP and subsequently exposed to the UVA light. The time of photoactivation is automatically calculated by the instrument based on the volume, the hematocrit of lymphomonocytic fraction, and the residual power of the UVA lamps (12). Finally, treated cells are reinfused back to the patients.

The first device approved for ECP treatment by the United States Food and Drug Administration (FDA) in 1988 was a closed system UVAR XTS (Mallinckrodt Pharmaceuticals, NJ, USA) (13). National approvals followed (14). The Therakos Cellex (Cellex), a second commercially available closed system device from the same manufacturer, was approved in Europe in 2008, Canada in 2009, and the United States in 2009. Advantages of the Cellex include smaller extracorporeal volume and shorter procedure time (15, 16). The Cellex system offers the ability to perform both single and dual needle procedures, further decreasing the run time and extracorporeal volumes (17). New technical developments and advances have substantially shortened the cycle duration and qualified ECP for use in patients with smaller body weight (14). Published reports have demonstrated the safety of the Cellex in the paediatric patient population with use of a blood prime (15, 16). There are no recommended quality control procedures to assess the collected product for Cellex device. Recently, a new closed protocol has been developed on the Amicus™ Separator (Fresenius Kabi, Germany) that enables the device to perform “in-line” ECP procedures when used in conjunction with the Phelix photoactivation device and associated disposable kit (18).

The “off-line” method was developed in 1994, following modification of the ECP procedure by Andreu et al. (19). The leukapheresis is performed using a standard continuous flow cell separator. Under sterile conditions, the collected cells are transferred to an appropriate receptacle, in which the 8-MOP is added and after UVA irradiation, cells are reinfused into the patient, using a standard transfusion set (19). This method was found to be safe, reproducible, and effective and is becoming more common in Europe than in USA, where only the “in-line” method is available (11).

At present, various devices are used in an “off-line” method. Cell separation and collection from peripheral blood is performed using standard apheresis devices such as Spectra Optia^®^ (Terumo BCT, USA), Com. Tec^®^ (Fresenius Kabi, Germany), MCS^®^ plus (Haemonetics, USA) and Amicus (Fresenius Kabi, Germany), all of which are FDA approved only for cell collection. Various photoactivation systems are used for UVA irradiation of cells, but none have been approved by FDA: PUVA combi light system (Cell-Max GmbH, Germany), MACOGENIC and MACOGENIC 2 (Macopharma, France), and UVA PIT system (MedTech Solutions, Germany).

Although the individual components may be Communauté Européenne (CE) marked, some devices used are not explicitly approved for use together in the process of photopheresis. Since the sterile barrier could be compromised when adding 8-MOP, it must be added in a background environment at least equivalent to GMP grade D, as required by directive 2006/86/EC. The open “off-line” technique can only be used by centres that are certified for cell therapy. The entire ECP procedure, including the drug and material to be used, transport, quality controls, traceability and the cell manipulation should be validated.

### Considerations before commencing ECP therapy

ECP requires long-term patient commitment, and many practical and clinical factors should be considered before starting. Practical factors include proximity of the patient to the treatment centre for feasibility of frequent treatments, as well as the suitability of peripheral venous access or risks of inserting a central venous catheter (10). Clinical variables to be assessed before each ECP procedure include complete blood count, and electrolytes calcium, magnesium and potassium in case ACD-A is used as an anticoagulant (10). Other pre-treatment blood tests may be considered on an individual basis for general assessment of the patient and/or the status of their GvHD (20). Regarding WBC, most centres consider that at least 1 × 10^9^/L cells in the peripheral blood are required before initiating the ECP session (11). Though there are still no data defining an adequate treatment dose of buffy coat and how that relates to peripheral WBC count, this WBC threshold is reasonable based on extrapolation of patient data from ECP-specific literature regarding collection efficiency kinetics and clinical outcomes (11, 21, 22).

As mentioned before, collection of cells using apheresis technique requires good venous access. The vascular access for ECP should be safe and efficient to allow a successful procedure over a long period of time and minimise the risk of infection and other complications including minimal interference with the patients’ daily life (11). Peripheral venous access should always be the first choice, but in patients with low body weight, children, and those with problematic venous access, permanent venous access devices with a proper blood flow are recommended. An apheresis-compatible central venous device with a double lumen may be required since an already existing central venous line often does not provide adequate blood flow. Permanent vascular access devices such as tunnelled cuffed central venous catheter and implantable ports allow long term ECP treatment (10).

### Contraindications to ECP

Absolute contraindications to ECP treatment include any known sensitivity to psoralen compounds, aphakia due to significantly increased risk of retinal damage, pregnancy, and uncontrolled infection (11, 13). Other conditions that could constitute at least relative contraindications are unstable circulatory or respiratory condition, cytopenia and low body weight (10). Haemodynamic instability due to sepsis or heart failure, or positive blood cultures would result in the cancellation of ECP (11, 23). In patients with a history of heparin induced thrombocytopenia, citrate should be used as anticoagulant. Precautions should be taken in patients with low haematocrit, low platelet count, active bleeding or risk of bleeding, and adequate transfusion support should be considered.

### Quality assessment of ECP product

Although quality assessment of ECP product is not required, the ASFA ECP subcommittee survey revealed that 34% of responding centres routinely perform quality control testing on the cellular product prior to re-infusion, using a variety of laboratory parameters (11) (**Table 1**). Quality assessment should be performed during “off-line” ECP for the first two sessions, and upon change of UVA illuminator or cell separator. Irradiation may be assessed by evaluating a change in the number of apoptotic 7-aminoactinomycin positive CD3+ cells within 72–96 h post-ECP (24). Currently, inhibition of T-cell proliferation after ECP is analysed using time-consuming assays including radioactive thymidine assays or carboxyfluorescein succinimidyl ester (CFSE) staining (25). Surface CD71 analysis represents a simple quality control alternative to detect ECP-mediated T-cell proliferation inhibition (26).

**Table 1:** Laboratory parameters routinely assessed on collected ECP product for quality assessment purposes [adapted from (11)].

Laboratory Parameter	Percentage Assessment
**Total cell count**	83%
**Haematocrit**	76%
**Lymphocyte count**	73%
**Monocyte count**	61%
**Bacterial culture**	24%
**Flow cytometric assay**	12%
**Apoptosis assay**	10%
**Evaluation of cell populations**	7%
**Proliferation assays**	7%
**Cytokine levels**	2%

### Side-effects of ECP

When compared to other immunosuppressive therapies currently available for the treatment of cGvHD, ECP is not associated with organ toxicities, the occurrence of opportunistic infections, treatment emergent adverse events or underlying disease relapse (14). The safety profile of ECP is excellent. Regardless of the device used, ECP is usually well‐tolerated with only uncommon mild side‐effects and no long-term complications. Analysis of data on 13,871 ECP procedures reported to World Apheresis Association (WAA) during a 17-years period showed that adverse events (AEs) were registered in 5.4% of first treatments and in 1.2% of subsequent procedures. Severe AEs were present in 0.04% of all procedures and no patient died due to the ECP procedure (27). Adverse reactions can be related to leukapheresis, such as transient hypotension caused by blood volume shift in the extracorporeal circuit, citrate toxicity due to anticoagulant used, mild anaemia and thrombocytopenia after multiple treatments, or bleeding from the cannula sites used for venous access. Reactions related to exposure to psoralen can include increased urinary output, metallic taste, increased skin redness or itchiness 6–8 hours after treatment and possibly some light sensitivity. On reinfusion of the ECP products, some patients complain of mild fever 2–12 hours after treatment, tiredness, and haematuria due to reinfusion of red blood cell postexposure to 8-MOP (9).

### Challenges, advantages, and disadvantages of ECP techniques

The two ECP techniques have not been directly compared in a clinical setting to date, but each pose their own characteristics (**Table 2**), challenges, advantages and disadvantages.

**Table 2:** Characteristics of “in-line” and “off-line” techniques.

Characteristic	“In-line” technique	“Off-line” technique
Principle	Closed single unit system	Open system with a separate device for each step
Apheresis technique	Continuous or discontinuous flow	Continuous flow
Venous access	Single or double	Double
Inlet flow (mL/min)	15-30 mL/min	30-60 mL/min
Anticoagulant	Heparin or Citrate	Citrate
Number of collected cells	Low	High Higher collection efficiency of lymphocytes
Breach of sterility	No	Yes: in 2nd step during dilution with saline and addition of psoralen
Clean room required	No	Yes
Quality control of irradiated cells	No	Yes: microbiological, haematologic, immunologic testing
Issues	Cost of photopheresis procedural kit	Centre must be certified for processing cell therapies Cost of use of clean room Risk of microbiological contamination Risk of infusion of irradiated cells to wrong patient
Duration	90-120 min	240 min

In “off-line” methods, apheresis devices offer higher collection efficiency of lymphocytes resulting in higher purity and numbers of harvested cells, which can be collected in less time and with low concentration of anticoagulant exposure. However, a higher cell collection has not previously been associated with increased therapeutic response. In a closed system with full integration and automation, there is no risk of improper reinfusion. The risk of infection or contamination associated with the medical device is therefore very low (14). In contrast, the “off-line” ECP procedure carries the risk of misidentification of cellular products during collection and processing, and procedures to prevent this situation must be in place.

Another advantage of the closed “in-line” method is that it lasts up to 120 minutes compared to the “off-line” method which takes twice as long, so it interferes less with other therapy that needs to be administered and reduces the workload of cell processing laboratory.

Although the treatment schedules and assessment of GvHD for children do not differ from the recommendations for adults, paediatric populations bring many unique issues to the ECP treatment. The performance of conventional ECP can be particularly challenging in young children, because of factors such as low body weight with limited extracorporeal volume, adequate vascular access, maintenance of intravascular fluid balance, patient’s tolerance of the lengthy procedure, and psychological implications (28). Blood prime of the apheresis device significantly reduces this risk of hypotension and haemodilution and is recommended in patients with low body weight.

Experimental modifications of the conventional ECP procedure have been developed, involving ECP-treated allogeneic cells, ECP treated enriched or depleted cell populations, cryopreservation of ECP-treated cells and mini-ECP (29). Mini-ECP is an alternative “off-line” technique that has been developed for the treatment of small children and patients with apheresis contraindications. In mini-ECP, usually 10 mL of blood per kilogram of the patient’s body weight is drawn from a central venous catheter and collected into a blood bag with anticoagulant, while the collected volume is simultaneously replaced with saline infusion. Buffy coat is extracted from whole blood using various devices such as: density gradient centrifugation with Lymphoprep (Nycomed, Norway), blood component extractor Compomat G4 (Fresenius Kabi, Germany), Spectra Optia apheresis system using Bone marrow processing program (Terumo BCT, USA) and automated laboratory separator Sepax (Biosafe, Switzerland) (30–33). Buffy coat is diluted with saline solution, 8-MOP is added, and the cells are irradiated by UVA illumination device. Irradiated cells are reinfused to the patient, but plasma obtained after the buffy coat separation is usually discarded and not infused back, because of its expected high cytokine content, as they are associated with the development of acute GvHD (34). Mini-ECP is usually performed according to the same treatment schedule used for conventional ECP. The results of all previously mentioned studies showed that the positive effect of mini-ECP was achieved despite the lower dose of treated cells than in conventional apheresis ECP (30–33).

An additional challenge facing ECP treatment is the availability of facilities equipped for ECP and the substantial cost associated with this long-term therapy (35, 36).

## Immunological mechanisms of ECP action

### Organ response to ECP and impact on systemic immunosuppression

Due to the complexity of organ involvement in cGvHD, it is challenging to assess the individual organ response, which is especially demanding in cutaneous cGvHD, where various sclerotic and non-sclerotic co-located lesions can occur in one patient. Based on a small number of prospective studies, the response to ECP should ideally be assessed after 6 months (6, 37). In a phase II randomised study by Flowers et al. with a weekly ECP regimen vs. standard therapy, the response to treatment was measured with total skin score (TSS) in 10 body regions at 12 weeks. At least a 25% decrease from baseline was observed in 8.3% of the ECP arm and 0% in the non-ECP arm, and the rate of complete responses (CR) and partial responses (PR) was significantly higher in favour of ECP (6). The response to prolonged therapy in 24 patients from this study group was reported by Greinix et al, with progressive improvement in TSS, 31% CR/PR in cutaneous cGvHD, and 70% CR/PR in mucosal symptoms at 24 weeks (37). In another prospective study by Foss et al, 20 (80%) out of 25 patients enrolled in the study achieved a regression of skin pathology, and in 6 (24%) patients healing of oral ulcers was noted. In 2019, Jagasia et al. published the results of a randomised, prospective study to investigate ECP added to standard of care (SoC) first-line therapy in cGvHD, based on the 2015 NIH consensus criteria for diagnosis and response assessment. Overall response rate (ORR) was 74.1% vs. 60.9% in the SoC+ECP arm vs. the SoC arm, with no decline in quality of life (QoL) in the arm treated with ECP (38).

Several retrospective studies have been published in which the authors attempted to grade the organ-specific response to ECP. In 2017, the UK Photopheresis Society published the consensus statement based on an updated review of the literature. The authors identified 27 studies with 725 adult cGvHD patients, with the mean response rate of 74% for skin involvement (23 studies), 62% for hepatic cGvHD (15 studies), 62% for mucosal cGvHD (12 studies), 60% for ocular cGvHD (4 studies), 46% for gastrointestinal (GI) cGvHD (5 studies) and 46% for lung involvement (9 studies) (39). Nevertheless, the role of ECP in pulmonary cGvHD is controversial. In a study by Del Fante et al, no response was observed in lung involvement (40). In a systematic review of prospective trials, the pooled ORR for pulmonary cGvHD was only 15% (0-50%) based on 3 studies (12 patients). In the remaining sites, the pooled ORRs were similar to those reported by Alfred et al: 71% for the skin (4 studies; 70 patients), 58% for hepatic involvement (3 studies; 45 patients), 63% for oral mucosa (3 studies; 32 patients), 62% for GI (2 studies; 9 patients) and 45% for musculoskeletal involvement (2 studies; 9 patients) (5). However, in a systematic review by Malik et al, the pooled ORR for pulmonary cGvHD was 48%, with comparable rates reported for the involvement of other sites (41).

### Use of ECP for steroid refractory cGvHD

Corticosteroids (CS) remain the best first-line treatment for cGvHD, and no concomitant immunosuppressive therapy (IST) was found to be better than steroids alone (42). However, in the case of CS therapy failure that occurs in about 50% of patients, steroid intolerance or steroid dependence, other agents may be used such as mycophenolate mofetil (MMF), ECP, mTOR inhibitors and others (42). The main advantage of ECP is the steroid-sparing effect, which has been reported by several investigators (6, 37, 43, 44) (**Table 3**).

**Table 3:** ECP in treatment of steroid-refractory chronic GHD. Adapted from (45).

Study	Trial Design	Treatment	ORR%/CR%	Other Results
Flowers et al., 2008 (6) (n=95; 48 vs 47)	Prospective, randomised, multicentre	ECP + CS+/- other IS vs CS+/- other IS	40 vs 10 at w12 in skin (p=0.002)	ORR in eye 30% vs 7% (p = 0·04) and mouth 53% vs 27% (p = 0·06); median % improvement of TSS at week 12 14·5% vs 8·5%, at week 24 31·4% in the ECP arm. ≥50% reduction in the CS dose in 17% of patients
Greinix et al., 2011 (37) (n=29)	Prospective, crossover, multicentre	ECP + CS +/- other IS	31 at w24 in skin	ORR in liver 50%, mouth 70% and joints 36%; median % improvement of TSS at week 24 25·8%. ≥50% reduction in the CS dose in 33%
Foss et al., 2005 (46) (n=25)	Prospective, single centre	ECP + CS + other IS	64/na	ORR in skin 80%, mouth 46%, joints 50%, lung 1 patient 50% improvement in DLCO, GI 33%, ocular 60%; CS sparing or IS discontinuation 80% (CS 44%)
Dignan et al., 2014 (47) (n=27)	Prospective, single centre	ECP +/- CS +/- other IS	70/7.4	ORR in lichenoid skin 72%, sclerodermatoid disease 80%, mouth 36%, ocular 54%; CS reduction 89.5% (17/19) including 26% CS discontinuation
Del Fante et al., 2012 (40) (n=102)	Retrospective, single centre	ECP +/- CS +/- other IS	80.5/15.7	In 9/16 CR patients IS was discontinued: the median prednisone change from baseline -77.6% (mg/kg)
Jagasia et al., 2009 (48) (n=64)	Retrospective, single centre	ECP +/- CS +/- other IS	58/11	decrease in CS doses (mean dose pre-ECP, 0.52 mg/kg versus 0.37 mg/kg post-ECP, p=.009).
Dignan et al., 2012 (49) (n=82)	Retrospective, single centre	ECP + CS +/- other IS	79/na	ORR in skin 92% and mouth 91% at 6 mo; 3-yr OS 69%. CS reduction 80% (40/50) including 22% CS discontinuation
Couriel et al., 2006 (50) (n=71)	Retrospective, single centre	ECP + CS +/- other IS	61/20	ORR in skin 57%, liver 71% and mouth 78%; 1-yr OS 53%; response to ECP and platelet count at ECP start significantly predict NRM. CS discontinuation 27% (12/59)
Nygaard et al., 2019 (51) (n=54)	Retrospective, single centre	ECP +/- CS +/- other IS	63/2	decrease in CS doses (from 25 to 10 mg prednisolone median dose) and 6 patients off CS
Apisarnthanarax et al., 2003 (52) (n=32)	Retrospective, single centre	ECP +/- CS +/- other IS	56/22	ORR in lichenoid skin 53%, sclerodermatoid disease 59%, visceral involvement 56%. 64% CS-sparing response rate
Ussowicz et al., 2013 (53) (n=13)	Retrospective, single centre	ECP +/- CS +/- other IS	69/na	CS-sparing in 84.6% (discontinuation in 6, substantial reduction in 5 patients)
Sakellari et al., 2018 (8) (n=88)	Prospective, single centre	ECP + CS	73/40	ORR in skin sclerosis 83%, visceral involvement 53% and lung 27%; 5-yr TRM 24%; 5-yr OS 64·5%
Gandelman js et al., 2018 (43) (n=77)	Prospective, multicentre	ECP + CS +/- other IS	62/14	ORR in skin 55%; ECP responses independent of risk factors of poor outcome
Greinix et al., 2006 (54) (n=47)	Retrospective, single centre	ECP + CS +/- other IS	83/na	CR in skin 68%, mouth 81% and liver 68%

ECP, Extracorporeal photopheresis; ORR, Overall response rate; CR, Complete response rate; CS, Corticosteroids; OS, Overall survival; TRM, Transplant related mortality; Pts, Patients; Mo, Months; HR, Hazard ratio; SR, Steroid-refractory; cGvHD, Chronic graft-versus-host disease; NRM, Non-relapse mortality; FFTF, Freedom from treatment failure; GI, Gastrointestinal; yr, Year; TSS, Total skin score; IS, Immunosuppressants; w, Week.

Focusing on the CS sparing effect of ECP, few prospective studies have been published. In the randomised phase 2 study by Flowers et al, ECP and conventional IST was compared with conventional IST alone in 95 patients with cutaneous manifestations of cGvHD that could not be adequately controlled by corticosteroid treatment (6). ORR at week 12 in skin was 40% in the ECP arm compared to 10% in the control arm (p = 0.002). Moreover, the percentage of patients experiencing both a 50% or greater reduction in daily corticosteroid dose and a 25% or greater improvement in the total skin score (TSS) at week 12 was higher in the ECP group than the control group (8.3%; 4 patients vs 0%; 0 patients; p=0.04). Best ORR was observed in oral mucosal involvement with 53% in the ECP arm and 27% in the control arm, respectively. At week 12, the median targeted symptom assessment scores improved in the ECP arm by 19% compared with 2.5% in the control arm (p=0.01) (**Table 3**). Since ECP does not induce general immunosuppression, risk of infections compared with other IST is not increased (55). In the latter study, infections were observed in 18% of patients in the ECP arm and 16% in the control arm (6).

In a follow-up open-label crossover ECP study by Greinix et al, 29 patients with lack of improvement or progression of SR cGvHD after 12 weeks of conventional IST were studied, with patients serving as their own controls (37). Significantly more patients in the ECP study compared with the initial non-ECP period achieved an ORR (CR or PR) of the skin (26% vs 8%, p=0.04), oral mucosa (65% vs 27%, p=0.009) and ocular involvement (27% vs 7%, p=0.04) at week 12 after crossing over to ECP treatment (37) (**Table 3**). Another prospective study by Foss et al. reported steroid-sparing or discontinuation of IST possible in 80% of patients (46). Similar results were reported by Dignan et al, in which among 19 patients who completed 6 months of ECP, in 17 (89.5%) CS dose was reduced, including 5 patients who discontinued CS (26%) and 8 (42%) patients with ≥50% reduction in the CS dose (47) (**Table 3**).

Several retrospective reports were also published. The study by Del Fante et al. includes 102 patients, with 76 patients treated with CS at baseline. In 9 out of 16 patients with CR, IST was discontinued, and in the CS-treated patients the median prednisone change from baseline was -77.6% (mg/kg) (40). In 43 patients treated by Jagasia et al, ECP led to a significant decrease in CS doses (mean dose pre-ECP, 0.52 mg/kg versus 0.37 mg/kg post-ECP, p=.009) (48). The study by Dignan et al. summarizes the results of ECP in mucocutaneous cGvHD, where 41 out of 53 (77%) patients who completed 6 months of ECP had IST reduction. Among 50 patients evaluable for assessment at 6 months, 40 (80%) had a CS dose reduction (including 11 who stopped, 19 ≥50% reduction) (49). Couriel et al. reported discontinuation of CS at 6 months in 12 out of 59 (27%) patients treated with CS at the time of ECP (50). In the longitudinal assessment of Nygaard et al, the median prednisolone dose was 10 mg/day (range 0–50) at the end of ECP treatment in 47 patients, including 6 patients off prednisolone (51). In the study by Apisarnthanarax et al, 28 patients treated with systemic CS at ECP initiation yielded a 64% steroid-sparing response rate with a median of 5.3 months of therapy (52). Among 13 patients with cGvHD described by Ussowicz et al, ECP enabled CS discontinuation in 6 and substantial dose reduction in 5 (84.6%) patients (53) (**Table 3**).

In September 2021 the Janus kinase (JAK) 1/2 inhibitor ruxolitinib was approved by the FDA for steroid refractory (SR) cGvHD, based on the results of the REACH-3 study (56). In the REACH-3 study, a randomised, open-label, multicentre clinical trial was performed comparing ruxolitinib to the best available therapy (BAT) for SR cGvHD. A total of 329 patients received either ruxolitinib at 10 mg twice daily or BAT including but not limited to ECP, MMF, MTX, rituximab, everolimus, sirolimus, and ibrutinib (56). ORR at week 24 according to National Institutes of Health (NIH) criteria (57) was 50% for the ruxolitinib arm and 26% for the BAT arm (p < 0.0001) including 7% and 3% CR rates. Ruxolitinib led to longer median failure-free survival (FFS) (>19 months vs 6 months, p < 0.001) and higher symptom response at week 24 according to the Lee symptom scale (24% vs 11%, p = 0.001). The probability of maintaining a response at 12 months was 68.5% in the ruxolitinib arm compared with 40% in the BAT arm. In the BAT arm, the majority of patients received ECP (35%, n=55) in addition to immunosuppressive therapy and achieved an ORR of 29.1% (PR=27.3%, CR 1.8%). Of note, ORR to ruxolitinib in liver and lung were 24% and 9%, compared to 22% and 6% in the BAT arm. Thus, treatment response also with ruxolitinib is still unsatisfactory. The NRM and relapse rate was similar in both treatment arms.

A retrospective analysis including 23 patients with SR cGvHD (57% NIH grade 3, 91% beyond second-line treatment and 87% with more than one organ involved), assessed the combination of ruxolitinib at 5 -10 mg bid and ECP every two to four weeks (58). Thirty-five percent of patients started ECP and ruxolitinib treatment simultaneously, whereas 30% started ECP at a median of 3.25 (range, 1-7) months before ruxolitinib. During ECP alone the best response was PR in 43% (3/7) of patients and 57% (4/7) were non-responders. Thirty-five percent of patients started ruxolitinib treatment first a median of 15 (range, 1-29) months prior to combination treatment. Best ORR to ruxolitinib alone was PR in 62.5% (5/8) and 37.5% (3/8) did not respond. Best ORR of ECP combined with ruxolitinib was 74% (17/23) including 9% CR and 65% PR and a two-year OS of 75% (58). Thus, combinational treatment substantially increased ORR in heavily pre-treated patients with multiorgan involvement SR cGvHD and was able to improve outcome of patients after inadequate responses to ECP or ruxolitinib monotherapy (58) (**Table 3**).

Due to changes in staging and response assessment of cGvHD according to NIH criteria (44, 57) and different primary endpoints (ORR at week 24 in REACH-3 vs median percent change in total skin score at week 12 in the phase 2 ECP study) the above mentioned randomised trials on the use of ruxolitinib or ECP in SR cGvHD cannot be reliably compared. Furthermore, REACH-3 enrolled only patients in need of second-line therapy of SR cGvHD and not more advanced ones and ruxolitinib was the only systemic intervention allowed at enrolment. In contrast, in the ECP study patients could also receive MMF and had a longer duration of cGvHD prior to study enrolment.

Overall, the steroid-sparing effect of ECP and excellent safety profile, including preserving antimicrobial immunity and not affecting the relapse risk, gives ECP the established level of recommendations (C-1; II) as the second-line treatment, according to the Consensus Conference on Clinical Practice in cGvHD (53, 59, 60).

### The interface between ECP and regulatory T cells in the context of cGvHD

**Figure 2:**
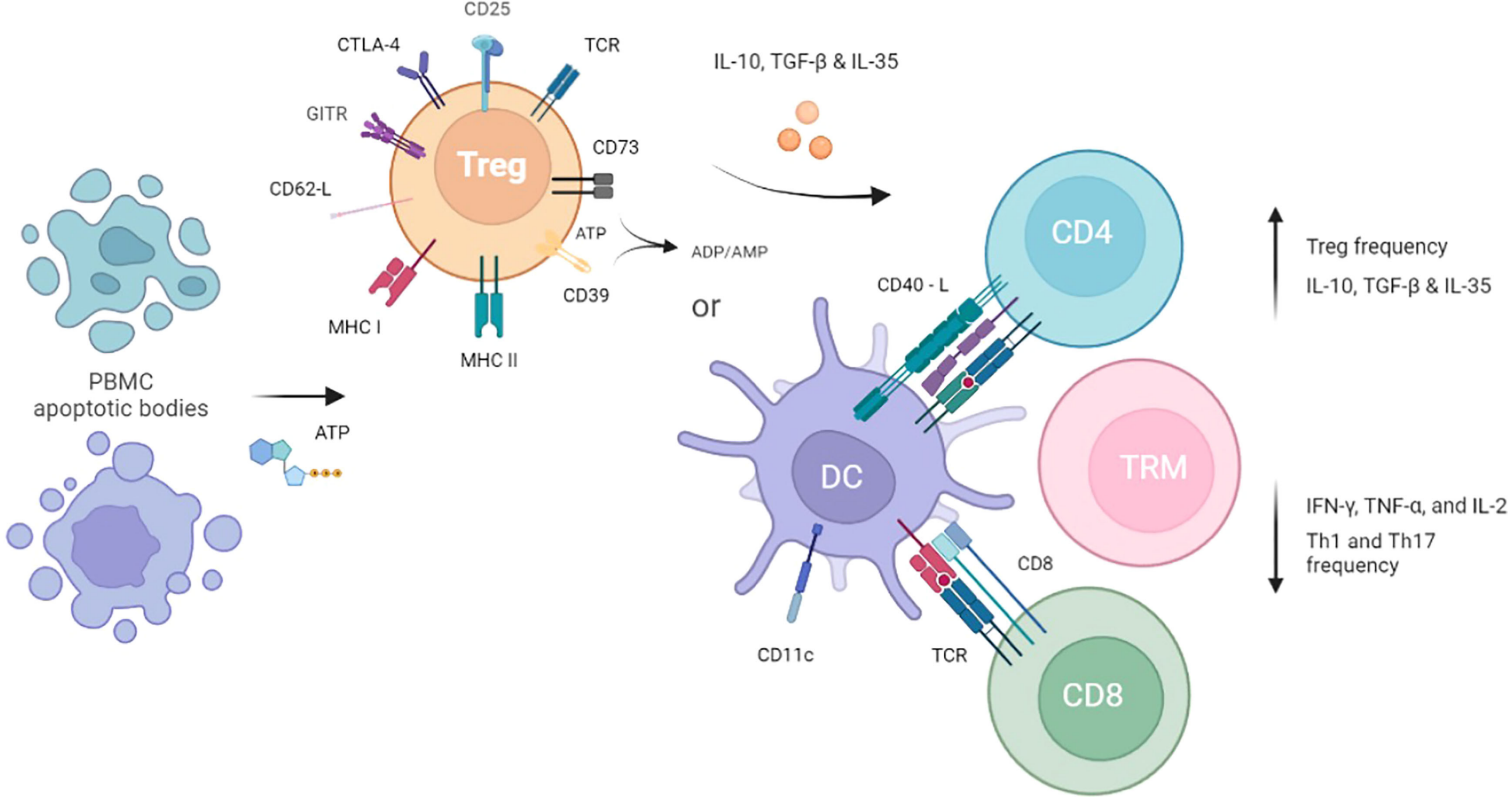
Hypothesised mechanism of ECP and tolerogenic immune cells immunosuppressive effects. The effect of ECP is first seen in the blood compartment where a portion of PBMCs go into apoptosis. Apoptotic bodies released by the PBMCs circulate and interact with Tregs as well as tissue-resident cells such as DCs, leading to tolerisation. Immunosuppression is achieved through cytokine excretion and cell-to-cell contact with mediators of GvHD such as CD4+ T helper cells, CD8+ T cells, and Tissue Resident Memory T cells.

Regulatory T cells (Treg) are a natural subset of CD4^+^ T cells essential for the establishment of peripheral tolerance and immune homeostasis. Tregs are mainly identified by the constitutive expression of CD25 (interleukin-2 receptor α-chain) and the transcription factor Foxp3, which is a key regulator for Treg development and function (61–63). Although Foxp3 is a specific marker for mouse Treg cells, it is well known that it can be induced in non-suppressor T cells upon activation in humans (64–66). Thus, other markers have been proposed to help discern conventional T cells from Treg, such as the low or absent expression of CD127 (interleukin-7 receptor) concomitant with higher levels of CD25 in Treg (67, 68). Regulatory T cells suppress different immune cell types through several mechanisms (**Figure 2**), which include modulation of antigen-presenting cells’ (APC) co-stimulatory markers *via* CTLA-4, competition for IL-2 and APC ligands, cytolysis, metabolic disruption by adenosine generation and transfer of cAMP, and secretion of anti-inflammatory cytokines (IL-10, TGF-β, IL-35) (69). The ability of Tregs to enforce peripheral tolerance, and suppress Th1 cell responses, has been shown to be partly *via* non-autonomous gene silencing (70, 71). This may be mediated by microRNA-containing extracellular vesicles; small, bilipid membrane-bound nano-vesicles that are released into the circulation and can act as intercellular communication molecules. Indeed, Treg cells are profuse producers of EVs that contain high levels of miRNA, the profile of which is distinct from Th1 and Th2 cells (70, 71). Treg-derived EVs can transfer specific sets of miRNA to conventional T-cells, both *in vitro* and *in vivo*. Compromised transfer of Treg cell EV miRNAs to conventional T-cells (either *via* failed miRNA formation (Treg cell Dicer deficiency), or inhibited EV release (Treg cell Rab27a- and Rab27b-deficient Treg cells)) has been shown to abrogate the capacity of Treg cells to prevent disease in a colitis model, by specifically regulating Th1 cell responses (70, 71). Furthermore, the profile of Treg EVs has been shown to be distinct from their parental cells, with enrichment of chemokines and interleukins (71). The specific role of Treg cell EV mRNA and proteins in modulating target cells in a context-dependant manner remains unknown, but seems likely to play a crucial role in their immunomodulatory effects (70).

Regulatory T cells have a fundamental role in autoimmunity, chronic inflammatory diseases, and transplantation. In the setting of haematopoietic stem cell transplantation, several studies demonstrated that the occurrence and severity of graft versus host disease (GvHD) can be associated with reduced levels of Foxp3^+^CD4^+^CD25^+^ Tregs (72–75). Furthermore, high donor Foxp3^+^ Treg content in the graft is associated with a lower risk of developing GvHD, further supporting the theory of impaired Treg homeostasis in patients with this complication post-transplant (76–78). Since the homeostasis of Treg is impaired in patients developing cGvHD, the level of Treg function has been intensely evaluated in this setting. In the context of ECP, several studies have shown that ECP treatment increases the frequency and activity of Treg cells in cGvHD patients (79–83), suggesting a critical role of Treg in the photopheresis curative process.

The first experimental evidence of apoptotic cells derived from ECP mediating antigen-specific Treg induction was observed in a murine model of photopheresis, through contact hypersensitivity (84). Injection of 8-MOP/UVA-irradiated leukocytes from sensitised mice inhibits immune response in recipient mice, in a process dependent on CD11c^+^ cells. This suppression could be adoptively transferred to a second generation of animals by Treg cells. Importantly, IL-10 derived from the 8-MOP/UVA-exposed cells was required for the ECP-mediated Treg effect (85). Moreover, studies from the same group have shown that DNA damage induced by UV radiation can promote immunotolerance by activation of antigen-specific Treg (86). It has been postulated that the ECP procedure impairs antigen presentation of the dying APCs, favouring Treg rather than effector T-cell activation (**Figure 2**). In fact, it has been demonstrated that human dendritic cells (DC) from ECP product are in an immature state and produce significant amounts of IL-10 (87, 88).

*In vivo* mouse models of GvHD aided the initial demonstration of the association between ECP, Treg and the resolution of the disease. First, it was reported that apoptotic cell infusion promotes TGF-β-dependent Treg expansion, which contributed to protecting mice from GvHD (89). Then, Gatza et al. extended those findings by incorporating ECP-treated cells in experimental GvHD, which reverted the established disease through an increase in CD4^+^CD25^+^Foxp3^+^ Treg frequency and *de novo* Treg generation, while indirectly reducing allogeneic responses of donor effector T cells (90). Overall, these early data in mouse models prompted other researchers to investigate whether Treg has a relevant role also in ECP-treated patients. The evaluation of Treg frequency in the initial phases of ECP could be useful to predict the final response and to evaluate the necessity of schedule intensification. Indeed, initial studies found a positive correlation between Treg numbers and activity with patients treated with ECP for organ transplants and type 1 diabetes (88, 91–93).

Regarding the ECP treatment for GvHD, Biagi et al. studied ten GvHD patients and found a significant increase of circulating Treg frequency compared to non-ECP-treated individuals, that peaked in cycles three and six out of ten ECP treatments (80). Besides the high expression of GITR, CD62L, CD45RO and Foxp3, these induced Treg were capable of suppressing IFN-γ production by CD4^+^CD25^-^ in a cell contact-dependent manner. In a follow-up study of the same group, enrolling more patients with a longer follow-up, their results suggested a correlation between CD4^+^CD25^+^Foxp3^+^Treg increase at the sixth cycle and clinical response only in cGvHD patients, which were compared to ECP non-responding controls and acute GvHD patients this time (81). Higher levels of Tregs in cGvHD patients with a complete or partial response to ECP therapy were also associated with decreased frequency of proinflammatory Th17 cells.

Considering the nature of the ECP procedure, in which less than 10% of leukocytes are exposed to the photosensitizing agent 8-MOP plus UVA, it is unlikely that these few apoptotic cells would be fully responsible for the clinical efficacy (90). Thus, a proposed mechanism of action involves further modulation of lymphocytes not exposed to ECP by the apoptotic cells after their reinfusion, thus inducing an immunotolerant environment rather than broad immunosuppression (94). It is also noteworthy that some studies indicate the clearance of apoptotic cells generates APCs with a tolerogenic phenotype (88, 95–97), leading to decreased stimulation of effector T cells and augmented production of anti-inflammatory cytokines, such as IL-10 (98–100). All these effects promote an immunotolerant milieu that favours Treg expansion and, subsequently, GvHD control. It is also relevant that Treg appear to be more resistant to *in vitro* 8-MOP plus UVA-induced apoptosis than other cell types (101). A direct influence on Treg activity involves the elevated extracellular ATP levels as a result of ECP-mediated cell death, which can, in turn, activate the CD39 and CD73 ectonucleotidases expressed by Treg (**Figure 2**). Indeed, an increase in CD39^+^ Treg cells frequency (102) and their adenosine production and ATP degradation were detected after ECP treatment in GvHD patients (103).

Nevertheless, the positive correlation between ECP treatment, Treg frequency and clinical improvement has not been consistently observed throughout the studies due to several reasons. There are major differences between studies regarding sample size, Treg markers, data analysis, and timepoints for peripheral blood collection. Besides that, the inter-patient variability is significant and may occur within the same cohort, namely the underlying disease before the transplant, the type of conditioning regimen and GvHD prophylaxis, the emergence of infections, the extent of tissue injury, the intrinsic heterogeneity of GvHD and disparities in the ECP protocol. All these parameters can play a role in the results obtained. For instance, a small study with eight steroid-refractory cGvHD patients searched four different phenotypes of Treg and found that all of them were increased in absolute numbers, but not in frequencies, three months after ECP compared to pre-treatment levels and healthy individuals (104). Although the three patients with the largest Treg increase had a reduction in daily steroid doses, no statistical correlation with clinical response was found, probably because of the small sample size. Similarly, several studies could not find a statistically significant correlation between a positive clinical outcome and augmented Treg cell frequency, suggesting that regulatory T cells may not be the dominant mechanism in ECP after all (43, 79, 105, 106).

Taken together, the somewhat controversial findings of Treg induction by ECP treatment strongly implies that future studies should focus on other aspects of regulatory T cell roles. Firstly, measuring only the percentages of circulating Treg may not be entirely sufficient to validate their importance to the ECP process, due to cell migration to other sites, especially to inflammatory tissues. Thus, whenever possible, the impact of Treg in target organs should also be addressed since it can be more qualitative than quantitative. Secondly, and since Treg do not have unique markers for identification, it is likely that different cell populations with divergent functional profiles were included in the Treg fraction of most studies. It is currently well established that the co-expression of CD4 and CD25 alone are not sufficient to identify classical regulatory T cells without knowing their methylation and functional profiles, since a large fraction of CD4^+^ cells expressing intermediate levels of CD25 are not suppressive. Thus, and even though it is difficult to isolate enough Treg from patients with cGvHD, it is critical to thoroughly evaluate the functional profile of circulating ECP-induced Treg. This can be achieved by sorting these cells and testing *in vitro* their capacity to suppress effector T-cell proliferation and cytokine production, as well as by disclosing their transcriptomic profile at the single-cell level.

### Immunomodulatory effects of ECP on circulating vstissue-resident immune cells in GvHD

The development of GvHD with the contribution of different immune cell subsets (e.g., circulating versus resident, donor origin versus host-derived) to tissue inflammation and the reversal of these processes by ECP remain unclear. In the course of HSCT, various myeloid and lymphoid immune cells undergo several dynamic stages before achieving steady state months after HSCT. Most circulating immune cells are depleted by myeloablative conditioning regimens, while tissue-resident cells are often not reached by these therapeutic strategies and contribute to post-transplant clinical courses and complications including GvHD (107, 108).

Tissue-resident memory T cells (TRM) are major contributors to adaptive immunity and can protect against pathogens at their entry site. However, these cells can also contribute to the initiation and propagation of (auto-) inflammatory diseases, such as GvHD (108, 109). The movement of T cells in and out of tissues involves the balance between tissue-retention proteins and tissue egress molecules on T cells. Tissue residency of T cells has been identified by different approaches in experimental animal models, including (i) parabiosis, in which mice are surgically conjoined and the origin of cells can be identified by genetic markers in the two partner mice (110, 111), (ii) mixed bone-marrow chimeras, in which mice are lethally irradiated and reconstituted with bone-marrow cells from congenitally marked animals (112, 113), and (iii) *in vivo* fluorescent antibody labelling to distinguish circulating from tissue-resident immune cells, the latter of which are protected from antibodies that are injected intravenously (114, 115). These studies revealed that TRM are generated upon infection with pathogens and that murine CD8+ and CD4+ T cells reside within barrier organs, which are commonly affected by GvHD. Currently accepted phenotypic markers for the identification of TRM from circulating T cells include CD69 and CD103 (116–118).

It was recently shown that the clinical course of GvHD is influenced by an interplay of tissue-resident T cells of host origin and donor-derived immune cells migrated from the circulation. We and others showed that a large percentage of immune cells in GvHD tissue are actually host-derived TRM, adding to the complexity of this process (108, 119). Recently, the concept of permanent tissue localization of TRM was challenged in murine experimental models that describe retrograde migrating cells with a TRM phenotype to draining lymph nodes, the blood and distant tissue sites (120, 121). This opens the interesting possibility that inflammatory T cells from affected organs initiate the inflammatory cascade at distant sites. Indeed, host-derived TRM can exit the skin after hematopoietic stem cell transplantation upon inflammation and contribute to GvHD in distinct organs (122). Transdifferentiation and entry into distant tissues by GvHD-driving TRM may represent a novel systemic factor contributing to this inflammatory multi-organ disease. By emigration from the skin into the circulation, these cells can become targetable by ECP and could be directly influenced by this systemic immunotherapy. However, indirect effects of ECP on TRM are also conceivable by interaction of resident immune cells with cell types that had been stimulated by ECP before migrating into tissues of GvHD-affected organs.

ECP as immunosuppressive second line treatment of GvHD has effects on a variety of immune cells and can induce Treg, as previously described (see chapter “The interface between ECP and regulatory T cells (Treg) in the context of cGvHD” of this review). It also affects effector T cells that might become or derive from TRM. Effector CD4+ T cell lineages can be classified into Th1, Th2, Th17/Th22 and Tfh cells according to their transcriptional, cytokine and chemokine receptor profile (123). While Th2 cells are found in acute GvHD, a Th1/Th17 signature can be observed in chronic GvHD (124). Long-term ECP therapy of acute and chronic GvHD exerts immunomodulatory effects on various effector T cells by promoting immune reconstitution and immune tolerance of T-helper cell subsets (**Figure 2**) (125).

The observation of changes in effector T cell lineages could be caused directly by ECP or mediated *via* other cell types that influence T cells within the tissue, where cellular interactions and cell-to-cell contacts are provided (126) and TRM are localised. In fact, maturation of monocytes to DCs facilitated by ECP is regarded as one of the mechanistic cores of ECP (127). Therefore, ECP is also regarded as monocyte-/myeloid cell-based cellular immunotherapy instrumental for adaptive immunity and tolerance mediated by various T cell subsets (128). Myeloid cells comprise a heterogenic group of cells that can differentiate from monocytes including DCs, macrophages and monocyte-derived suppressor cells. Dendritic cells are professional antigen-presenting cells (APC) that sense antigens in barrier tissues, migrate to lymphoid organs to prime naïve T cells becoming tolerogenic or immunogenic antigen-specific effector cells. Consequently, DC induced by ECP are instrumental for antigen-specific T cell-mediated immunity and tolerance (129). The change in DC morphology and function has been demonstrated in several different diseases treated by ECP and is likely to be the primary biological effect of the treatment (**Figure 2**). Additional effects of ECP occur downstream from dendritic cell changes and reflect the disease process that is being treated, the age of the patient and extent of organ damage. ECP, through the action of DCs, induces a change in the cytokine environment from a pro-inflammatory Th1 to an anti-inflammatory Th2 response, with a decrease in IFNγ, TNFα, and IL-2 secretion and an increase in TGFβ serum levels (130). In addition, monocyte-derived macrophages are important for tissue homeostasis and take a centre stage in inflammatory disorders (131). Monocyte-derived suppressor cells can directly influence the disease course of GvHD (132) or act *via* regulatory T cells (133, 134). Specifically in the context of ECP, there is also a mechanical effect on mononuclear cells due to the movement of blood through plastic tubing. Monocyte and dendritic cell differentiation and maturation changes have been demonstrated after blood has flowed through plastic tubing, probably mediated *via* activated platelet signalling (9).

The interaction of human circulating and tissue-resident T cells with monocyte-derived DCs is crucial to understand mechanistic insights of ECP. To investigate the shift of effector T cell lineages upon ECP and the influence of ECP-stimulated monocytes on T cell subsets, a human *in vitro* model of ECP was developed. This co-culture model provides evidence that ECP-treated monocytes reduce proliferation of CD4+ T cells and change their cytokine secretion pattern in a contact-dependent manner (135). This is an example of how T cells – including TRM – can be influenced by other ECP-targeted cells in GvHD patients.

### Naive T-cells/TRECs in ECP treated paediatric patients

As previously discussed, the age of the patient is also an important determinant to the final outcome of ECP treatment. In the HSCT setting, paediatric patients have the ability to completely reconstitute normal immunity, as thymic function is still present, and donor stem cells are able to differentiate into donor stem cell-derived, but recipient thymus educated T cells, using central thymic tolerance mechanisms to reduce allo-reactivity (136). However, the primary mode of action appears to be directed at DCs, through uptake of apoptotic mononuclear cells, initiating a tolerogenic rather than pro-inflammatory milieu (100), promotion of Tregs (80) and upregulation of peripheral tolerance mechanisms.

In paediatric patients with aGvHD, the increase in peripheral tolerance leads to a reduction in the inflammatory effect of donor stem cell-derived, donor thymus educated T cells. This is reflected in the resolution of symptoms of aGvHD in skin, liver and intestine, but also in the inflammatory damage to the thymus. In combination with the concurrent reduction in conventional immunosuppression, particularly cortico-steroids, thymic recovery is promoted. One small study of paediatric patients treated with ECP for aGvHD demonstrated emergence of new thymic emigrants (determined by a rise in T cells containing T cell receptor excision circles, a DNA marker of thymopoiesis), with an increase in diversity of the T-lymphocyte receptor. These changes were accompanied by changes in metabolic pathways favouring Tregs and inhibiting effector T cells (137).

## Current biomarker studies for ECP response in cGvHD

Given the heterogeneous nature of GvHD, prediction of risk, prognosis, and treatment response monitoring would be of significant benefit, allowing for a more personalised treatment plan for patients following HSCT. Successful biomarkers offer objective, unbiased information on systemic disorders, and significant effort has been made to identify effective biomarkers for GvHD. Ideally, a GvHD biomarker should be actionable, utilizing the results of biomarker testing to guide clinical management of disease and clinical trial design. Biomarkers may be used for diagnostic or predictive reasons, define response to therapy or guide prognosis and risk assessment. However, to date no GvHD biomarker has been accepted for clinical use (138). Biomarker identification and validation in cGvHD is not straightforward, due to the heterogenous impact on recipient organs, increased timeframe of onset and course of the disease and lack of multicentre trials with sufficient patient samples (138, 139). Additionally, age related differences in the biology of cGvHD may exist (139, 140). Further research is necessary to establish how GvHD biomarkers are best incorporated into ECP treatment pathways, with the goal of tailoring ECP to the needs of individual patients and maximizing ECP benefit (141).

### Clonal T cells

One of the first studies on biomarkers for ECP response in GvHD investigated whether circulating clonal T cells in peripheral blood (PB) and clonal T cell receptor γ (TCRγ) rearrangement, could be linked to response to ECP, as was previously demonstrated in cutaneous T cell lymphoma (CTCL) (142, 143). Peripheral blood samples of 27 patients (extensive cGvHD n=17, no cGvHD n=10) were analysed and TCRγ gene rearrangements and amplified clonal T cell populations were found in 60% of the patients without cGvHD and in 76.5% of patients with cGvHD, compared to 0% of the healthy controls. Eight of 12 patients receiving ECP showed significant response and had amplified clonal T cell populations. The authors concluded that expanded clonal T cell populations in the patients with cGvHD before treatment significantly increased the probability of cutaneous response to ECP (143) (**Table 4**).

**Table 4:** Biomarkers for response of cGvHD to ECP (141).

Study	Cohort	Biomarker	Findings	Rational
French et al., 2002 (143)	27 pts. (extensive cGVHD n=17, no cGVHD n=10)	Clonal T cells, TCRγ	Increased circulating clonal T cells showed greater chance of response to ECP	Clinical responsiveness to ECP in CTCL has been shown to be dependent on the presence of detectable circulating clonal T cells in the peripheral blood
Kuzmina et al., 2009 (144)	49 pts. (with moderate n=25 or severe n=4 cGVHD)	Immature CD19^+^CD21^−^ B lymphocytes	Significantly higher proportions of immature CD19^+^CD21^−^ cells prior to ECP in pts. With no response to ECP after 6 months	Role of B lymphocytes in autoimmune diseases, and a role in the pathogenesis of cGvHD
Akhtari et al., 2010 (145)	25 pts.	Myeloid DC, plasmacytoid DC, CD4+, CD8+	higher baseline circulating DCs and T cells in ECP responders	Investigate an *in vivo* effect of ECP on numbers of circulating DCs and T cells in patients with cGvHD
Whittle and Taylor 2011 (146)	46 pts.	BAFF (B-cell activating factor)	Lower BAFF levels after 1 month of ECP predicted better response at 3 and 6 months	BAFF influences immature B-cell survival and promotes production of autoantibodies. Excess BAFF may contribute to cGvHD by protecting alloreactive/autoreactive clones from apoptosis. Elevated BAFF levels correlate with cGvHD activity
Bertani et al., 2016 (147)	15 pts	CD3+	CD3+ numbers in first three months of ECP correlated with subsequent clinical response	The number of lymphocytes collected and reinfused during ECP treatment might be associated with response to treatment
Montoya et al., 2019 (148)	10 pts. (n=31 cGvHD, n=14 aGvHD)	miR34a-5p, miR-148a-3p	Increased expression in GvHD pts prior to ECP compared to controls, decreased levels with ECP therapy.	Combined microRNA expression may act as circulatory biomarker for ECP response
Crocchiolo et al., 2021 (149)	12 pts	Regulatory T cells (Treg)	Patients that further developed cGvHD (n=5) had fewer circulating Treg counts during the 1^st^ month after prophylactic ECP than those who didn’t develop GvHD (7)	Immunomodulation mediated by ECP influences Treg cells
Lopes et al., 2020 (83)	6 pts	Regulatory T cells (Treg)	ECP responders had higher levels of circulating Tregs, most noticeable when ECP was continued for long periods (5-8 years)	

B cell activating factor; cGvHD, Chronic graft-versus-host disease; CTCL, Cutaneous T cell lymphoma; ECP, Extracorporeal photopheresis.

### B lymphocytes

Another study investigated levels of B lymphocyte subsets in 49 patients with moderate (n=25) and severe (n=24) cGvHD, measuring immature CD19^+^CD21^−^ B cells and memory CD19^+^CD27^+^ cells as prediction markers for ECP response (144). The proportions of memory CD19^+^CD27^+^ cells prior to ECP were not significantly different between the groups, however there was a significantly higher ratio of CD21− to CD27+ cells before treatment in patients showing no response. In addition, ECP non-responders at 6 months showed a significantly higher percentage of immature CD19+CD21− B lymphocytes (mean 22%) in PB before the start of ECP and at 6 months of ECP compared with CR (mean 8%) and PR (mean 16%) patients. Whether these findings are ECP-specific has to remain speculative, because no other treatment cohorts were analysed and the mechanisms of action of ECP are still subject to further research. Therefore, CD19+CD21− B lymphocytes could also serve as a biomarker for measuring disease activity of cGvHD (144) (**Table 4**).

### Plasma B-cell activating factor

In addition to B lymphocytes, plasma B-cell activating factor (BAFF) levels were also investigated in following studies. Whittle and Taylor used BAFF as a biomarker predicting ECP treatment response in 46 patients with cutaneous cGvHD (146). BAFF levels after 1 month of ECP predicted response at 3 and 6 months. Patients with BAFF concentrations of <4 ng/mL showed decreased skin cGvHD and complete resolution in 11 patients, while those with high BAFF concentrations showed worsened skin cGvHD at 6 months and resolution in only 1 patient. BAFF levels after 3 months of treatment predicted probability of maintaining improvement for another 3 months (146) (**Table 4**).

### T cells and dendritic cells

A subsequent study investigated correlation of response to ECP with patients’ baseline circulating DCs and T cells in 25 cGvHD patients (145). Patients who responded to ECP had higher baseline circulating myeloid and plasmacytoid DC precursors and CD4+ and CD8+ T cells, which can predict response to ECP in cGvHD patients. The study noted that apart from a decrease in CD4+ cells in responsive patients, there was no significant changes in CD8+ T cell of DC numbers over a 12-month period following ECP treatment (141, 145).

In a retrospective study focused on T lymphocyte population, including CD3+ harvested during ECP procedures (147), the treatment response to ECP of 15 patients with SR cGvHD was correlated with the amount of CD3^+^ lymphocytes collected. Analysis showed that CD3^+^ numbers collected with apheresis for ECP during the first three months of treatment were associated with clinical response (**Table 4**). This prediction of response may identify patients early on in treatment who are responding to ECP and exclude those who are unlikely to achieve clinical response (141, 147).

### Regulatory T cells

Recently, independent groups have suggested the potential use of Treg counts as a biomarker of ECP effectiveness (83, 149, 150). Of note, one study has shown that the increase in circulating Treg associated with ECP treatment in responding patients is most noticeable when ECP is continued for a long period of time, as the follow-up lasts for up to eight years (83). Another study explored administration of the ECP method as a preventive measure, which additionally raises another path for investigation. In fact, among twelve patients that underwent prophylactic ECP after HSCT, lower absolute numbers of CD4^+^CD25^+^CD127^-^HLA-DR^+^ Treg cells were found in the group of patients developing cGvHD (149) (**Table 4**). Thus, highlighting that modulating Treg homeostasis may be an important component of ECP success.

In the context of Tregs, Denney et al. examined CD4^+^CD25^+^CD127^dim/−^Foxp3^+^ Treg absolute counts and frequencies of 32 patients with cGvHD treated by ECP from three months up to one year (105). Compared to pre-ECP levels, both percentages and counts of Treg increased gradually at months six and nine after treatment started, although no statistical association with improvement in cutaneous cGvHD, global organ involvement and steroid tapering was found. There were similar results in a small study with 15 patients, in which CD4^+^CD25^bright^ cells percentages increased constantly with ECP treatment until the 18^th^ month in all cGvHD patients, regardless of their response to the photopheresis (106). Moreover, applying a logistic regression model, Gandelman et al. could not find a clear pattern for both CD4^+^CD25^+^CD127^lo^Foxp3^+^CD45RO^+^ Treg and cutaneous lymphocytes antigen (CLA)-expressing Treg frequencies between responders and non-responders of ECP in more than 30 samples analysed. Instead, Treg numbers were highly variable across the patients (43).

Interestingly, other groups have shown a positive correlation between Treg and ECP, but only in specific situations. In a clinical trial that combined ECP treatment with low-dose IL-2 for cGvHD patients, Treg absolute counts and Treg : Tcon cell ratio only increased after IL-2 supplementation, not exclusively in responders (151). On the other hand, aligned with the concept that Treg levels are affected by different clinical variables, Zhu et al. encountered a trend for higher CD4^+^Foxp3^+^ Treg percentages only in ECP-responders cGvHD patients without a history of both aGvHD and sclerosis, the fibrotic form of cGvHD (152); however, the reduced number of samples compromised statistical analysis. On the contrary, the previously cited work of Denney et al. could not find significant differences in Treg parameters between the 22 patients that had prior aGvHD and the 10 that did not (105). Nevertheless, those studies are important to highlight that the different clinical manifestations of GvHD should be taken into consideration when addressing the ECP effects.

### MicroRNAs

More recently, microRNAs have been assessed as potential biomarkers in the context of GvHD. These small (~22 nucleotide), non-coding RNAs function to downregulate translation of mRNA and have been shown to possess critical regulatory functions in virtually all physiological processes, targeting up to 50% of the human genome (153). MicroRNAs may be considered as ideal biomarker candidates, as they are aberrantly expressed in many disease states, present in non-invasively collected biofluids, highly stable and their collection and assessment can be performed using clinically translatable methods. Several authors, including ourselves (154–156), have identified microRNAs that target key pathways implicated in the pathology of GvHD. However, our understanding of the role of microRNAs in the mechanisms of ECP action as therapy for GvHD, as well as biomarkers for ECP outcome, is still relatively understudied.

In a pilot study conducted by Montoya et al, an initial cohort of 10 GvHD patients (7 cGvHD and 3 aGvHD) demonstrated increased pre-ECP plasma expression of miR-22-5p, miR-34a-5p, miR-148a-3p and miR-505-3p compared to healthy controls, which decreased to comparable levels by 6 months post-ECP (148) (**Table 4**). Increased expression of miR-34a-5p and miR-148a-3p was validated in 45 independent GvHD patients (31 cGvHD and 14 aGvHD), whereby expression was increased in GvHD patients prior to ECP treatment compared to controls, and microRNA levels significantly decreased with therapy. Interestingly, this effect was not observed in an independent cohort of patients receiving ECP therapy for lung transplantation, indicating the effect was GvHD-specific and not intrinsically associated with ECP therapy. At 6 months post-ECP, patients responding to therapy demonstrated higher pre-ECP levels of miR-34a-5p, and lower miR-148a-3p expression compared to non-responders. This effect was further demonstrated by receiver operator characteristics (ROC), which also indicated that a combination of both markers showed a stronger effect than either microRNA alone (148). Although these results indicate the potential for miR-34a-5p and miR-148-3p as circulatory biomarkers for ECP response in patients with ECP, the authors did not report their effect in the individual subtypes of aGvHD and cGvHD. It would be interesting to validate their biomarker potential in cGvHD specifically, as well as explore the source of microRNA and their individual involvement in the mechanism of ECP response.

Also of biomarker relevance, it has been shown that in the context of cutaneous T cell lymphoma (CTCL), an early increase in expression of miR-191, miR-223 and miR-342 expression in PBMC at 3 months post-ECP was associated with clinical response at 6-12 months (157). Although the ECP process does not differentiate which disease is being treated, it is not clear if increased microRNA expression at 3 months in comparison to baseline is directly attributed to ECP therapy, or representative of altered levels in the context of underlying CTCL. These results therefore warrant further investigation in a post-HSCT setting.

## Conclusion

During the last years, efforts have been made to improve outcome of patients with cGvHD by new NIH consensus guidelines, new therapeutic approaches and combination therapies, but also by more in-depth research of the mode of action of ECP.

In published studies, ORR to ECP varies from 40% to 80%, with the highest organ responses reaching approximately 80% in the skin and mouth, 70% in the liver, 60% in the eye and 50% in the joint and visceral involvement. The role of ECP in pulmonary cGvHD is still controversial. However, even in patients with disease stabilization, ECP allows the reduction of CS doses, and in some patients with CR or PR, ECP enables discontinuation of IS. In patients with SR cGvHD, ECP has a steroid-sparing effect, as evidenced by reduction in corticosteroids concomitant with clinical improvement of cGvHD features. The combination of ruxolitinib and ECP in SR cGvHD not responding to either treatment alone has synergistic effects, with both therapies inhibiting proinflammatory signals. The importance of fully understanding the immunomodulatory mechanism of action of ECP is increasingly apparent, in order to better tailor personalised treatment schedules.

With regard to TRM and effector T cell subsets, we know that these play an important role for tissue inflammation during GvHD. They are also important targets for ECP, either directly or *via* interaction with monocyte-derived dendritic cells and/or macrophages. With newly applied T-cell-targeted therapies, TRM with their pattern of cytokine and immune checkpoint molecule expression have become a focus of immunotherapy. The interesting aspect exists that these novel experimental approaches can be combined with ECP in future for a more efficacious treatment with a beneficial profile of side effects. While the importance of Treg in ECP mechanism of action is increasingly recognized, it is essential to consider a broader assessment of Treg phenotype and function, instead of limiting the study to measurement of Treg cell frequencies. Further knowledge in this setting may indicate that a modulation of Treg activity synergizes with ECP response, thus leading to an improved prognostic. Crucially, it is also important to elucidate how much of the ECP response may be explained by spontaneous fluctuations in GvHD activity, as immunological changes in cell composition may be driven by ECP but also by GvHD itself and the effect of steroid withdrawal.

With the availability of easy-to-assess biomarkers, we are on the verge of developing new tools to monitor or even predict treatment success, in order to optimize management of cGvHD patients. Further research is needed to establish how cGvHD biomarkers are best incorporated in ECP treatment pathways with the aim of tailoring ECP to meet the needs of individual patients and maximizing benefit. This should incorporate larger cohorts of patients studied prospectively with longer follow-ups and well-defined clinical parameters, submitted to robust centralized *in vitro* analysis.

## Author contributions

RC designed and coordinated the preparation of the manuscript. IB, RC, AF, AG, HG, RK, JL, CP, AP, DP, KS, GS and NW planned content and wrote the manuscript. All authors contributed to the article and approved the submitted version.
